# Comment on Russo et al. Does Tumor Volume Have a Prognostic Role in Oropharyngeal Squamous Cell Carcinoma? A Systematic Review and Meta-Analysis. *Cancers* 2022, *14*, 2465

**DOI:** 10.3390/cancers14174283

**Published:** 2022-09-01

**Authors:** Gabriel Adrian

**Affiliations:** Division of Oncology and Pathology, Clinical Sciences, Skåne University Hospital, Lund University, 22242 Lund, Sweden; gabriel.adrian@med.lu.se

I read the article by Russo et al. [1] with great interest and acknowledge the authors’ attempt to investigate the prognostic role of tumor volume in oropharyngeal squamous cell carcinoma. The authors performed a meta-analysis and included data from 10 studies where some studies used dichotomized tumor volume (such as in [2,3]), and others used tumor volume as a continuous variable (such as in [4,5]) in their original analyses. The separate studies have shown significant importance of tumor volume both as dichotomized variable in [2,3] (*p* < 0.0001 and *p* = 0.0003, respectively) and as continuous variable in [4] (*p* < 0.001 (hazard ratio (HR) per unit tumor volume)). In the current meta-analysis, the authors calculated an HR of 1.02 for overall survival and 1.07 for loco-regional control. The authors claim that the effect size is too small to be clinically relevant.

As a reader, it is hard to fully comprehend how the pooled analyses were conducted and how to interpret the results. Firstly, the authors seem to have mixed HR for tumor volume as a continuous variable (HR_continuous_, i.e., the increased risk per cm^3^ tumor volume) and dichotomized tumor volumes (HR_dichotomized_, i.e., large compared with small tumor separated at a specific tumor volume). For instance, in Figure 2A the HR_continuous_ for Carpen et al. [5] denotes the risk per unit increase in tumor volume (e.g., 1.02 for p16-negative), which corresponds to an increased risk of 1.02 *per cm^3^ increase in tumor volume.* Thereby, comparing a tumor that is 40 cm^3^ to a tumor of 10 cm^3^, the estimated risk for OS-event increases by 1.02^30 = 1.81 (i.e., 81% increased risk). The Lok et al. [3] report used dichotomized data and found an HR_dichotomized_ of 3.74 for large vs. small tumors (threshold 32.79 cm^3^) among the 340 studied patients (who were assigned a weight of 0.2% despite constituting 45% of all included patients). 

Secondly, and perhaps a consequence of the first issue, the pooled analyses were weighted in an ambiguous manner. As depicted in Figure 4A, a weight of 35.7% was assigned to the 19 patients with p16-negative patients in Carpen et al. [5], whereas the 160 patients in the CF-arm of Adrian et al. [2] received a weight of 0.9%. Perhaps the authors relied on the absolute value of the standard error (SE) to assign weights to the cohorts? If that is the case, the weight for the studies using HR_continuous_ will be falsely inflated (since the numerical value of HR_continuous_ and corresponding SE are lower compared with HR_dichotomized_). The authors calculated a pooled HR of 1.02 for overall survival based on data from 760 patients. Additionally, 99.2% of the weights were assigned to the 278 patients reported by Panje et al. [4] and Carpen et al. [5], who both used HR_continuous_ in their analyses (with HR_continuous_ in the range 1.01–1.02 per cm^3^). An HR_continuous_ in that range could be highly clinically relevant, as exemplified above. 

In summary, the intention of the performed meta-analysis is of high relevance to the community, but the presented analyses are difficult to interpret. In fact, if the authors re-do the calculations separately for studies using HR_continuous_ and HR_dichotomized_, the conclusion may very well be that tumor volume is a reliable prognostic factor for oropharyngeal squamous cell carcinoma with high clinical significance.
